# Behavioral effects of mefloquine in tail suspension and light/dark tests

**DOI:** 10.1186/s40064-015-1483-8

**Published:** 2015-11-17

**Authors:** John Michael Holden, Richard Slivicki, Rachel Dahl, Xia Dong, Matt Dwyer, Weston Holley, Crissa Knott

**Affiliations:** Department of Psychology, Winona State University, Phelps Hall 230-B, Winona, MN 55987 USA; Indiana University, Bloomington, USA

**Keywords:** Mefloquine, Malaria, Emotion, Tail suspension, Light–dark box, Mice

## Abstract

Mefloquine hydrochloride has been used widely in the past few decades for malaria prophylaxis and treatment. However, in recent years, it has fallen out of favor due to reports of exposure being linked to numerous neuropsychiatric effects, including emotional disturbances. In this study we examined the effects of different doses (5, 25, or 100 mg/kg) of mefloquine relative to vehicle on male C57BL/6 J mice in two tests of emotional behavior, the light–dark box and the tail suspension test. It was found that mefloquine exposure reduced anxiety-linked behaviors in the light–dark box and reduced total immobility times in the tail suspension test, especially at higher doses. Our results lend support to the notion that mefloquine exposure could induce emotional disinhibition.

## Background

Mefloquine hydrochloride [dl-erythro-α-(-2-piperidyl)-2, 8-bis (trifluoromethyl)-4-quinolinemethanol], historically has been used as a prophylactic and treatment agent for malaria infection caused by *Plasmodium falciparum* (Nevin 2012a; Magill 2006). Mefloquine was developed at the Walter Reed Army Institute of Research in the 1970’s and had been marketed under the trade name Lariam, among others (Nevin 2012b). Due to its long half-life in the body (Yelmo et al. 2010), allowing for weekly rather than daily treatment, and its efficacy against chloroquine-resistant forms of the disease (Nevin 2012b), mefloquine was employed widely among travelers to areas where malaria infection was likely (Nevin 2012b; Petersen et al. 2000; van Riemsdijk et al. 1997, 2005; Phillips and Kass 1996) including members of the U.S. military deployed overseas to such areas as Iraq and Afghanistan (Nevin 2010; Peterson et al. 2011; Vuurman et al. 1996).

In the past 10–15 years, concerns about mefloquine’s neuropsychiatic side effects have grown. Initially it was believed that side effects were rare (1 in every 10,000 cases; Choo 1996). Yet, numerous case studies of travelers with (Wittes and Saginur 1995; Even et al. 2001; Peterson et al. 2011) and without complicating factors such as a prior history of psychiatric difficulties (Nevin 2012a; Yelmo et al. 2010) suggest that exposure to prophylactic regimens of mefloquine or higher treatment doses are associated with side effects, including insomnia (Yelmo et al. 2010), depression (Caillon et al. 1992; Javorsky et al. 2001, Whitworth and Aichhorn 2005), manic behavior and/or bipolar disorder (Piening and Young 1996; Yelmo et al. 2010), mental confusion and concentration difficulties (Javorsky et al. 2001; Nevin 2012a; Tor et al. 2006), and memory impairment (Javorsky et al. 2001; Nevin 2012a). Especially troubling are reports of symptoms indicative of mania and confusional psychoses, including symptoms of insomnia, elevated mood and energy, disinhibition in social interactions, delusions and hallucinations (Björkman 1989; Javorsky et al. 2001; Kukoyi and Carney 2003; Peterson et al. 2011; Piening and Young 1996; Stuiver et al. 1989; Tor et al. 2006), which have been shown to respond to treatment with antipsychotics (Piening and Young 1996; Tor et al. 2006). Suicide and suicidal ideation have been also noted (Jousset et al. 2010). Some case studies indicate psychiatric disturbance lasting long after treatment was withdrawn (Kukoyi and Carney 2003). Larger studies of travelers have also found evidence of depressed feelings, “strange feelings” and other alterations in emotional and cognitive functioning (Petersen et al. 2000; van Riemsdijk et al. 1997, 2005; Meier et al. 2004), especially among women (van Riemsdijk et al. 1997; Phillips and Kass 1996; Schlagenhauf et al. 2009).

The mechanisms by which mefloquine could potentially create adverse effects on mood and emotional functioning are numerous. Mefloquine has also been shown to affect gap junction activity by blocking the connexin36 protein (Nevin 2012c; Voss et al. 2009; Juszczak and Swiergiel 2009; Alisky et al. 2006), to alter dopaminergic and cholinergic activity, and calcium homeostasis (Juszczak and Swiergiel 2009; Alisky et al. 2006; Nevin 2011; Allison et al. 2011), to stimulate 5-HT2A and 5-HT2C receptors with similar potency and efficacy as the hallucinogen dimethyltryptamine (Janowsky et al. 2014), to alter activity in basolateral amygdala, important to the mediation of fear and anxiety states (Chung and Moore 2009), to impair fear-based learning through blocking of hippocampal gap junctions (Bissiere et al. 2011), to potentially alter sleep-waking related activity in reticular activating sites (Beck et al. 2008; Garcia-Rill et al. 2007), and to antagonize adenosine receptors (Alisky et al. 2006; Shepherd 1988). Rodent studies have found that mefloquine administration led to changes in sleep phase activity, motor function (proprioception), lesions in brain stem, especially the nucleus gracillis (Dow et al. 2006), and induced tonic seizures (Amabeoku and Farmer 2005). Thus, mefloquine has the potential to produce both acute and long-term deleterious effects.

Given the notable evidence of significant pharmacodynamic and toxicodynamic effects of mefloquine in the brain, it is surprising that so few studies have directly explored its behavioral effects. Considering the wide variety of symptoms mefloquine exposure has been linked to—elevated energy, insomnia, anxiety, confusion, social disinhibition, depression, manic-like and agitated psychotic symptoms, mefloquine may have a fundamental disinhibiting effect on emotional regulation—through its arousing, fear-related, and even hallucinatory effects and effects on neurotransmitters systems related to arousal, such as dopamine and adenosine—that could contribute to the emergence of a number of psychiatric syndromes. To further investigate the etiology of observed behavioral effects of mefloquine during clinical use, we explored the effects of mefloquine in a rodent model using two murine tests of emotional behavior: the light–dark apparatus and the tail suspension test. The light–dark apparatus (Bourin and Hascoët 2003; Keers et al. 2012; Flaisher-Grinberg and Einat 2010; Shoji et al. 2012) allows measurement of a number of anxiety related variables in mice. Mice are placed in an apparatus which gives them a choice of exploring a lighted area (which is explored less when the subject is anxious) or staying in a more secure, darkened compartment. We hypothesize that the acute administration of mefloquine would lead to a reduction in anxiety-related behaviors in the apparatus, due to its putative effects on emotional regulation.

The tail suspension test is a murine model of depressive-like behavior (Cryan et al. 2003; Stéru et al. 1987), in which mice are suspended by the tip of their tail for a short period of time (Xiaoqing and Gershenfeld 2001). This suspension typically leads to initial struggling and attempts to escape followed by increasingly lengthy periods of immobility. Drugs with an antidepressant effect, such as desipramine, tend to reduce the amount of time spent immobile in this task, as do stimulant drugs such as amphetamine and caffeine (Tenn et al. 2005). This test has been used to test for manic-like (Shoji et al. 2012; Kirshenbaum et al. 2013) as well as depressive-like behavior (Wang et al. 2014; Zhu et al. 2014), using time immobile as a measure of emotional behavior. We hypothesized that acute administration of mefloquine would decrease periods of immobility in this test; again, this would be a function of mefloquine’s effects on emotional regulation.

## Results

### Light/dark test

**Fig. 1:**
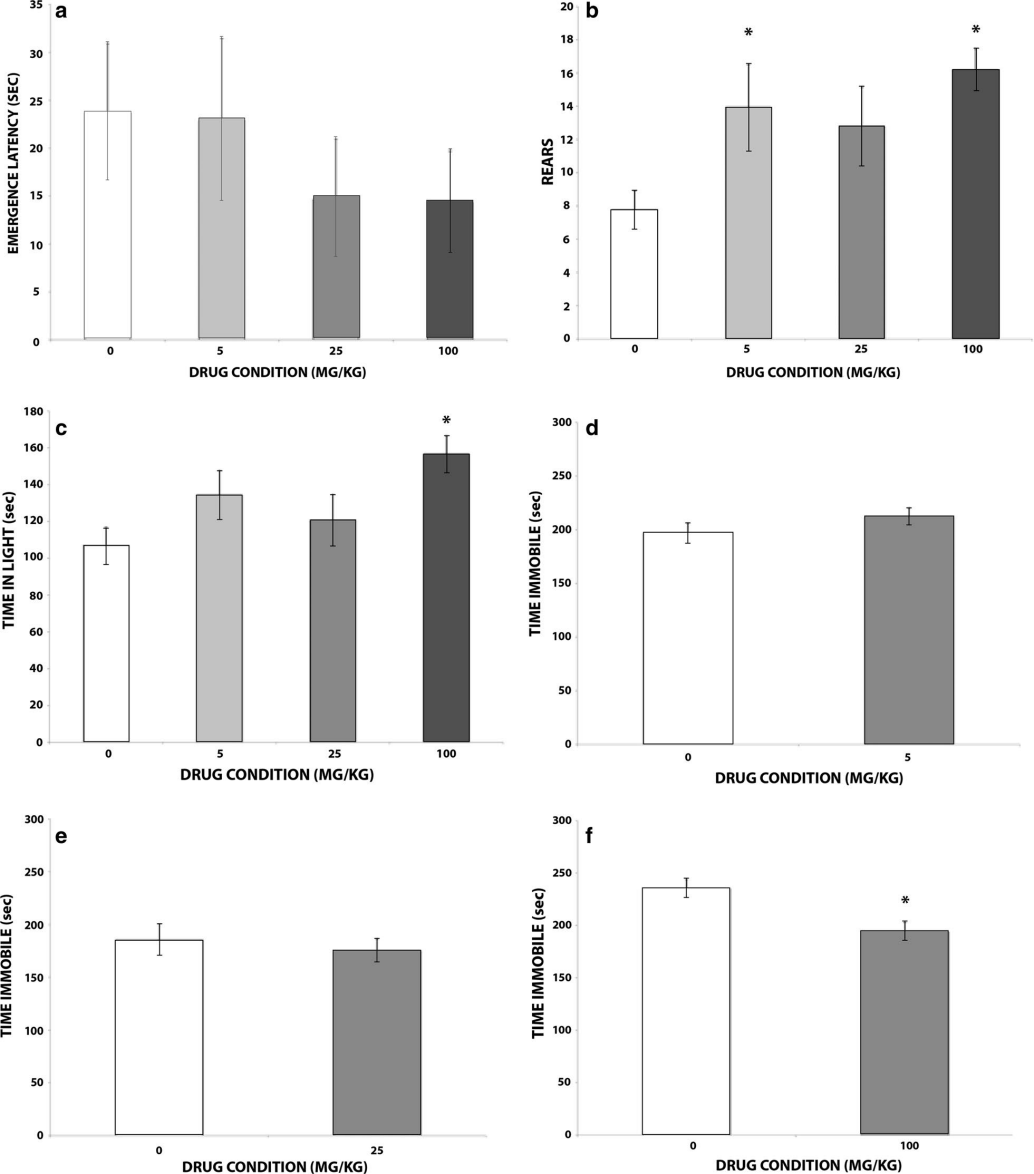
Behavior as a function of drug dose. **a** Emergence latency in the light dark test. **b** Rearing behavior in the light dark test. **c** Total time spent in the lighted area in the light dark test. **d** Total immobility time in the tail suspension test for Set 1 (0 vs. 5 mg/kg mefloquine). **e** Total immobility time in the tail suspension test for Set 2 (0 vs. 25 mg/kg mefloquine). **f** Total immobility time in the tail suspension test for Set 3 (0 vs. 100 mg/kg). All data is presented as *M* ± SEM. *Indicates a significant difference between indicated group and 0 mg/kg controls, *p* < .05

Figure 1a displays the emergence time as a function of drug dose (mean = 23.64, 22.89, 14.68, and 14.3 for 0, 5, 25, and 100 mg/kg, respectively). Although there was a general trend towards shorter emergence latencies, a one-way ANOVA demonstrated no effect of dose, *F*(3,68) = 0.468, *p* = .693. Figure 1b shows the number of rears by dose (mean = 7.77, 13.95, 12.79, and 16.18 for 0, 5, 25, and 100 mg/kg, respectively). A one-way ANOVA showed a significant effect of dose, *F*(3,68) = 3.946, *p* = .012. Fisher’s LSD showed that 5 and 100 mg/kg doses produced a greater number of rears than vehicle, *p* < .05 for both. Figure 1c illustrates the total time spent in the light in seconds (mean = 107.04, 134.35, 120.78, 156.55, for 0, 5, 25, and 100 mg/kg, respectively). A one-way ANOVA revealed a significant effect of dose, *F*(3,68) = 3.359, *p* = .024, with Fisher’s LSD further showing that the 100 mg/kg dose was significantly greater than vehicle, *p* < .05.

### Tail suspension test

Figure 1d–f illustrates the total time spent immobile in the tail suspension test by dose. Analysis of Set 1 (Fig. 1d) showed no significant difference between vehicle and the 5 mg/kg dose (mean = 196.72 and 211.85, respectively), *t*(23) = 1.165, *p* = .256. Analysis of Set 2 (Fig. 1e) showed no significant difference between vehicle and the 25 mg/kg dose (mean = 185.35 and 175.18, respectively), *t*(26) = 0.538, *p* = .298. Finally, analysis of Set 3 (Fig. 1f) showed significantly less immobility in the 100 mg/kg group relative to vehicle, *t*(27) = 3.054, *p* = .005.

## Discussion

The results of this study confirmed the hypothesis that administration of mefloquine would lead to the emergence of behavior in our subjects indicative of emotional disturbance. Subjects in the light/dark test showed an increase in rearing behaviors and time spent in lighted areas of the box at the highest dose employed, while changes in rearing behavior were noted at the smallest dose as well. Emergence latency was highly variable and no significant differences were seen, although a general trend downwards with increasing doses was suggested. Moreover, a decrease in immobility was seen in the tail suspension test with the highest dose, further suggesting the occurrence of an altered emotional state; lower doses, however, had no effect on immobility. This study provides converging evidence consistent with case reports of emotionally disinhibited behavior associated with mefloquine exposure (Yelmo et al. 2010; Piening and Young 1996).

Greater rearing and time spent in the light in the light/dark test has traditionally been interpreted as indicating lower levels of anxiety (Bourin and Hascoët 2003), and decreased immobility in the tail suspension has typically been interpreted as indicating an anti-depressive effect (Wang et al. 2014; Zhu et al. 2014). Our data is not incompatible with these possibilities. However, as previously discussed, mefloquine’s effects on the brain are many. Greater activity could be indicative of anxiolysis, given mefloquine’s effects in fear-mediating areas of the brain (Bissiere et al. 2011; Chung and Moore 2009), or it could be indicative of greater arousal due to effects on adenosine (Alisky et al. 2006; Shepherd 1988), serotonin (Janowsky et al. 2014), dopamine (Allison et al. 2011), or effects on sleep-waking systems (Beck et al. 2008, Garcia-Rill et al. 2007). The present study on its own, while supporting the idea of a fundamental emotional disinhibition, cannot determine whether the behavioral effects seen are indicative of anxiolytic, anti-depressive, or arousal based effects. Other studies have yielded mixed results regarding the question of mefloquine’s effects on arousal and activity; for example, Dow et al. (2006) found that mefloquine changes some measures of activity in rats, such as beam traversal time and time spent active during the sleep cycle, but not others, such as in the open field test. Future research should address these separate possibilities.

The importance of this work is evident when considering all the complicating factors in determining mefloquine’s effects in humans, and especially travelers who may be experiencing other events—illness, exhaustion, adjustment to a new situation and/or culture—that could complicate determinations of causality. Randomized and double-blind studies of travelers do exist (Schlagenhauf et al. 2009), but typically rely on participant-provided retrospective reporting across a long period of time. The features of a controlled study on a homogeneous subject population with direct measures of behavior employed in an exact time frame, rather than retrospective reports gathered after a trip abroad, are especially germane to determining the short-term effects of mefloquine. Many reports of adverse effects indicate that such effects arose shortly after the consumption of the first dose, whereas reporting of adverse effects by travelers may take place several weeks later.

Of relevance to the current work is the association of mefloquine with both depression and mania, both of which have been identified in case studies (Yelmo et al. 2010; Caillon et al. 1992; Javorsky et al. 2001; Whitworth and Aichhorn 2005; Piening and Young 1996). It is possible that mefloquine’s stimulatory effects—through antagonism of adenosine receptors (Alisky et al. 2006; Shepherd 1988) or other mechanisms—could produce manic-like behavior within a short time after administration. Moreover, due to mefloquine’s long-half life in the body, continued stimulation of adenosine receptors over the course of many days could continuously interfere with sleep, leading to symptoms of depressed mood rather than mania; indeed, insomnia is one of the most common adverse effects reported (Yelmo et al. 2010).

Despite these results, interpretation of the current study is somewhat complicated by dosing issues. While a range of doses was tested, it is unclear which of these doses employed best represents a dose given to humans for prophylaxis or treatment. In one study of rats, the oral doses of mefloquine in corn oil that produce concentrations similar to that seen in humans after prophylaxis and treatment are 45 and 187 mg/kg, respectively, 24 h after administration (Dow et al. 2006); however, direct comparisons with that study are complicated by differences in species and route of administration. As such, this study cannot definitively establish that the dose of oral mefloquine given to humans for prophylaxis (250 mg weekly) or treatment (750 mg or greater) were equivalent to any of the doses used in this study with mice. It can be said, however, that the emergence of behavioral disturbances in our study was noticeable with greater doses, and as such, the risk of manic-like behavior is likely greater with the larger, treatment dose. This is worth noting in part because it would be difficult to determine that mefloquine was responsible for behavior disturbances if given to a person already assumed to have an active infection and likely to suffer post-malaria neurological symptoms (Nevin 2012a).

Further complicating understanding of mefloquine’s effects on behavior is the fact that mefloquine’s effects are idiosyncratic and influenced by the function of the P-glycoprotein transmembrane transporter, which mediates mefloquine’s movement across the blood–brain barrier (Nevin 2012d). Polymorphisms in the ABCB1/MDR1 gene coding for P-glycoprotein may account for individual differences in mefloquine accumulation in the brain, which in turn have been proposed to mediate mefloquine’s treatment efficacy with progressive multifocal leukoencephalopathy and might similarly influence individual differences in the behavioral effects of mefloquine exposure. Considerable differences in behavioral sequelae of exposure could result from individual genetic differences in influx and efflux of mefloquine from the brain.

It should also be noted that other murine tests not explored in this study could also be used to establish the relationship between acute exposure to mefloquine and emotional disinhibition, including the resident-intruder test (Einat 2007)—such an approach could also be valuable in elucidating mefloquine’s putative relation to aggressive behavior—and hedonia as measured by the sweetness preference test (Flaisher-Grinberg and Einat 2009). Studies of startle behavior could be helpful in disambiguating whether mefloquine’s effects on activity in the current study are indicative of anxiolysis or not. Should mefloquine have an anxiolytic effect, it should decrease startle magnitude; conversely, if mefloquine induces a more fundamental emotional disinhibition of the kind that that underlies confusional psychosis and mania, we should see exactly the opposite. A separate issue is whether the effects shown here are the result of mefloquine’s effects on adenosine or through some other mechanism; studies of co-administration with adenosine antagonists could be illustrative in this regard. In the future, we hope to incorporate the use of behavioral recording software to more precisely track behavior in these and other tests.

Currently mefloquine is considered a fourth-line agent for treatment of malaria in many regions (Nevin 2012e), in large part because of the risk of adverse effects identified previously. As such, the reported incidence of adverse effects associated with the drug could reasonably be expected to diminish over time as providers turn to better tolerated, safer alternatives. However, mefloquine is still being employed, in part because of established resistance to other antimalarials such as chloroquine; thus, it seems likely that the issue of potential adverse effects will still be relevant in the near future. Moreover, there remains the largely unexplored issue of long-term adverse effects associated with exposure. It remains to be seen how many of those exposed long-term to the drug will develop lasting issues, particularly members of the military deployed for years overseas. Research on longer term effects is especially important given the possibility of limbic encephalopathy (Nevin 2012a) lesions in brainstem and elsewhere (Dow et al. 2006), and longer-lasting manifestations of illness mediated by mefloquine exposure. Currently our lab is exploring the effects of longer-term exposure to the drug. Regardless, the current results support the hypothesis that even acute mefloquine exposure can induce symptoms of mood disturbance.

## Conclusions

Our study concludes that acute administration of mefloquine leads to some behaviors indicative of emotional disinhibition in mice, including increased rearing and time in light in the light/dark apparatus and reduced immobility in the tail suspension test.

## Methods

### Subjects

Subjects were 166 male C57BL/6 J mice, approximately 3–4 months old, bred from stock obtained from Jackson Laboratories (Bar Harbor, ME) and housed under standard conditions with free access to food and water under a reverse 12:12 h light:dark cycle with lights off at 10 AM. All procedures were approved by the Institutional Animal Care and Use Committee of Winona State University. Mefloquine hydrochloride (Sigma-Aldrich, St. Louis, MO; 5, 25, or 100 mg/kg body weight) in corn oil vehicle or vehicle alone was injected intraperitoneally 24 h before testing. Testing was conducted at approximately 4 PM–6 PM each day.

### Light/dark test

The light–dark test was conducted in a darkened room. The light–dark apparatus consisted of two chambers separated by a black plastic door. The first chamber (start box) was a 19 × 14.5 × 20 cm black plastic chamber (dark area), with a wooden door that was manually removed at the beginning of each test. Outside the doorway, there was a chamber measuring 50 × 39 × 20 cm with white-painted wooden walls and a floor made of white-painted wood, above which was a 60-watt bulb that illuminated the lighted part of the chamber. At the beginning of testing and in-between sessions, the apparatus was wiped clean using a 75.5 % ethanol solution. Subjects were handled daily for several days before the beginning of testing. Subjects were administered 0 (n = 22), 5 (n = 19), 25 (n = 14) or 100 (n = 17) mg/kg mefloquine 24 h before testing. Before testing, subjects were removed from the colony room to the darkened testing room for approximately 10 min. Subjects were then placed in the dark box for 30 s. At that point, the doorway between dark and lighted areas was opened and the test began. Three variables were assessed: (a) the amount of time required for each subject to emerge from the dark chamber into the lighted field (emergence latency, defined as the time after the doorway opened when all four paws entered the lighted field), (b) the number of rearing behaviors in the lighted area, and (c) the total time (out of 300 s) spent in the lighted field (light time). After a 5-min period, the subject was removed from the apparatus and the apparatus was cleaned with ethanol. Sessions were videotaped and scored by observers who were blinded to the experimental condition for each subject.

### Tail suspension test

The tail suspension apparatus for the acute test consisted of a plastic frame supporting a PVC tube (approximately 2 cm in diameter) approximately 15 cm from the ground. Clear tape was used to suspend the subjects from the tube by the tip of the tail, ensuring paws remained off the ground, even when outstretched. Subjects were handled daily for several days before the beginning of testing. Separate groups of subjects were run for each dose comparison, such that each group given mefloquine had its own separate comparison group. Although this approach potentially led to a loss of statistical power, as control subjects from each set could not be combined into a single large control group, it was deemed necessary since separate sets were scored by different observers. Set 1 subjects were given either vehicle (n = 19) or 5 mg/kg mefloquine (n = 18); Set 2 subjects, either vehicle (n = 14) or 25 mg/kg mefloquine (n = 14); Set 3 subjects, vehicle (n = 12) or 100 mg/kg mefloquine (n = 17). Subjects were then moved to an illuminated room and affixed to the tube by the tip of the tail with clear tape so that they remained suspended for 6 min. Sessions were videotaped and scored by observers who were blinded to the experimental condition for each subject. The variable of interest was the amount of time spent immobile (limbs not moving, trunk and head immobile) out of 360 s. Mice that climbed their tails during the test were eliminated from the project; 5 oil treated controls and 7 mefloquine treated mice from the 0 vs. 5 mg/kg comparison were eliminated for this reason.

### Statistics

Data are expressed as mean + SEM. All statistics were computed using SPSS. Normality of data was confirmed using the Shapiro–Wilks test. One-factor ANOVAs and Fisher’s LSD post hoc tests were used to compare groups for the light–dark test, and independent-samples t test (Student’s) were used to compare groups for the tail suspension test, with alpha levels set at .05.
